# Paraneoplastic Dermatomyositis Due to Gallbladder Carcinoma

**DOI:** 10.7759/cureus.97036

**Published:** 2025-11-17

**Authors:** Jugal James, Joe James, James Jose

**Affiliations:** 1 Radiology, Government Medical College, Kozhikode, Kozhikode, IND; 2 Neurology, Government Medical College, Kozhikode, Kozhikode, IND

**Keywords:** dermatomyositis, gallbladder carcinoma, gottron’s papules, paraneoplastic syndrome, upper limb weakness

## Abstract

Dermatomyositis is an inflammatory myopathy that can be sporadic or paraneoplastic. A 72-year-old female presented with a subacute progressive illness characterized by symmetrical proximal weakness of upper and lower limbs, dysphagia, and neck flexor weakness. On examination, she had erythematous, scaly lesions over the periorbital areas, face, and interphalangeal and metacarpophalangeal joints (Gottron’s papules). Neurological examination revealed bilateral palatal palsy, proximal predominant upper and lower limb and neck flexor weakness, areflexia with preserved knee jerks, waddling gait, and normal sensory modalities. Investigations showed polymorphonuclear leukocytosis and elevated creatine kinase. Electromyogram revealed short-duration, small-amplitude polyphasic potentials with fibrillations and positive sharp waves consistent with inflammatory myopathy. Antinuclear antibodies and myositis antibodies were negative. Contrast-enhanced CT revealed a polypoidal lesion in the gallbladder with liver metastasis. She declined a biopsy and was started on prednisolone with minimal improvement. The patient expired one month later.

## Introduction

Dermatomyositis is an autoimmune myopathy characterized by inflammation in the muscle biopsy with an incidence of 1 in 100,000 [1]. It is characterized by subacute, symmetrical proximal weakness of upper and lower limbs, often with involvement of neck flexors. Typical skin findings include heliotrope rash, Gottron’s papules, erythematous lesions over the upper chest (V-sign) and upper back (shawl sign), mechanic’s hands, and nailfold capillary abnormalities. Extramuscular manifestations include interstitial lung disease, arthritis, dysphagia, cardiomyopathy, and cardiac conduction abnormalities. An associated malignancy has been found in 9.4% cases of dermatomyositis [2,3]. The commonly reported malignancies include ovarian, lung, breast, nasopharyngeal, gastric, pancreatic, and colorectal cancers. Reporting dermatomyositis as a presenting sign of gallbladder cancer is important because recognition of this paraneoplastic syndrome can prompt targeted malignancy screening and potentially alter patient outcomes. Several reports document the regression of cutaneous/myopathic features treatment of the underlying gallbladder tumor. Here, we report a case of dermatomyositis secondary to gallbladder carcinoma.

## Case presentation

A 72-year-old female presented with pruritic and erythematous lesions around the eyes, neck, upper chest, arms, and legs for the past four weeks. One week later, she started to have dysphagia, which was more for liquids, with occasional tracheal aspiration of feeds, and a nasal twang of voice. Over the next two weeks, she developed proximal weakness of both upper limbs in the form of difficulty raising arms overhead, followed by lower limb weakness in the form of difficulty getting up from a chair. By this time, she also noticed difficulty in raising her head from the bed. There was no history of facial weakness, ptosis, diplopia, or sensory symptoms. She did not report any loss of weight, anorexia, fever, or other systemic symptoms.

**Figure 1:**
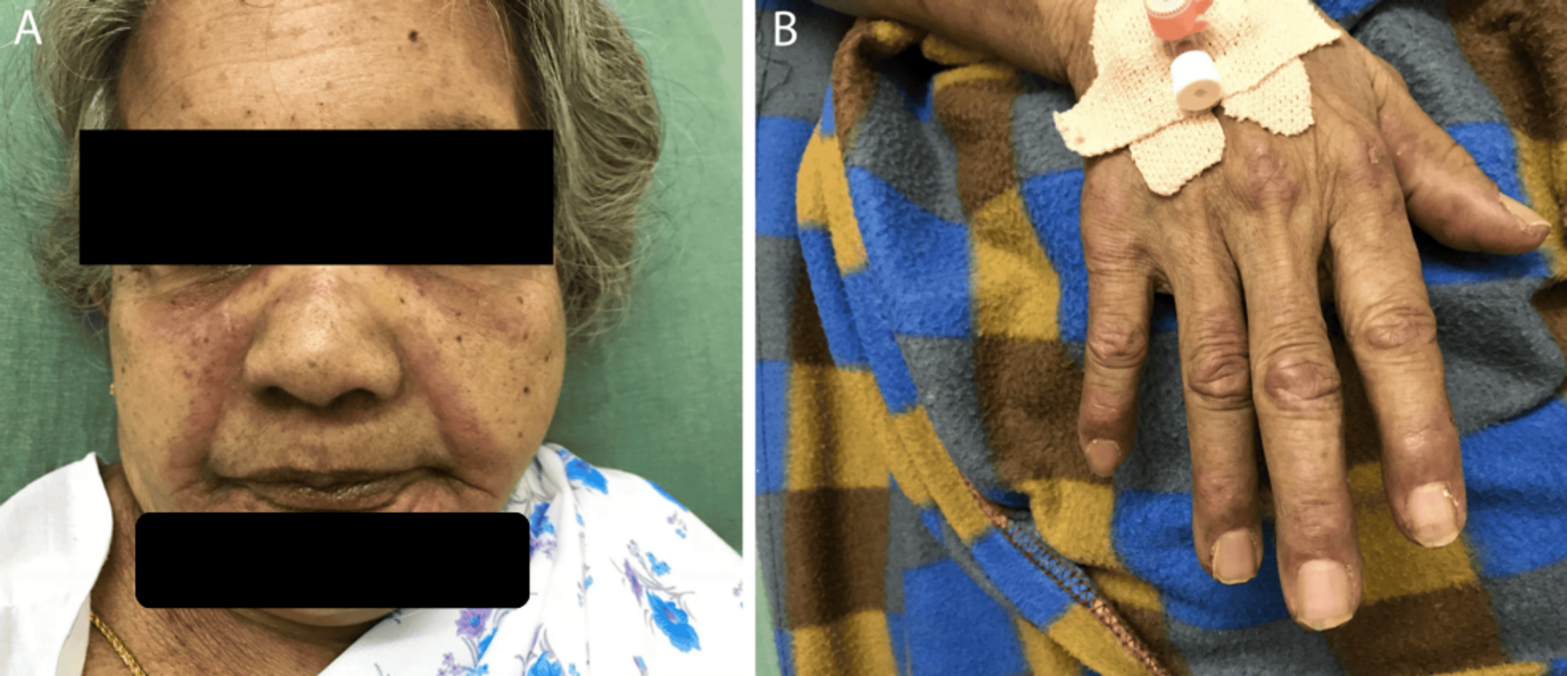
(A) Erythematous, scaly lesions over the periorbital areas, nasolabial folds, and lower jaw. (B) Gottron’s papules over the metacarpophalangeal and interphalangeal joints.

**Figure 2:**
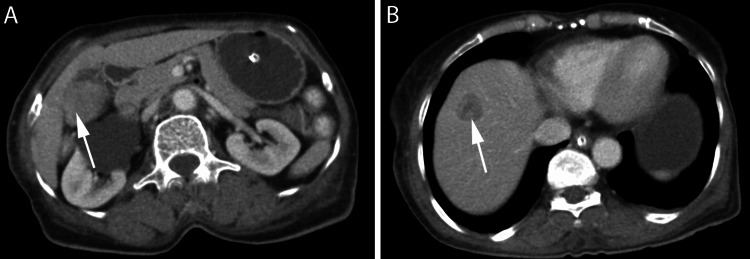
Contrast-enhanced CT revealing enhancing polypoidal soft tissue density lesion involving the gallbladder (A) with solitary liver metastasis (B).

On examination, she was alert and oriented. Vital signs were stable. Erythematous, scaly lesions were noted over the periorbital areas, nasolabial folds, lower jaw (Figure 1A), and over the extensor aspects of metacarpophalangeal and interphalangeal joints (Figure 1B). Generalized edema was noted over the face, hands, and legs. Cranial examination showed bilateral palatal palsy. Power was Medical Research Council Grade 1/5 in shoulders, Grade 3/5 in elbows, and Grade 4+/5 in wrists with mild handgrip weakness. In the lower limbs, power was Grade 3/5 in the hips and Grade 4/5 in the knees and ankles. Trunk and neck flexors were also weak. All deep tendon reflexes except knee jerks were absent, and plantar reflexes were flexor bilaterally. Sensory examination was normal. Blood investigations showed polymorphonuclear leukocytosis and a creatine kinase level of 7,200 U/L (normal: 39-238 U/L). Electromyogram revealed fibrillations and positive sharp waves (PSWs), with short-duration, small-amplitude polyphasic potentials consistent with myopathy. A clinical diagnosis of dermatomyositis was made. Antinuclear antibodies, myositis antibodies (anti-Mi-2β, Ku, PM-Scl-100, PM-Scl-75, Jo-1, SRP, PL-7, PL-12, EJ, OJ, RO-52) were negative. Chest X-ray and ultrasound of the neck were normal. Contrast-enhanced CT revealed a heterogeneously enhancing polypoidal soft tissue density lesion involving the gallbladder with solitary liver metastasis (Figures 2A, 2B). She declined a biopsy and opted for palliative care. She was started on steroids without much improvement and expired one month later.

## Discussion

The diagnosis of dermatomyositis was made in our patient with the typical presentation of symmetrical proximal weakness, characteristic skin rashes, Gottron’s papules, elevated creatine kinase, and myopathic electromyogram, even without a muscle biopsy. Moreover, the presence of fibrillations and PSW in a myopathic electromyogram is characteristic of inflammatory myopathy. Myositis-specific autoantibodies are found in about 30-50% cases of dermatomyositis, which were absent in our case [4]. Although the myositis panel we used included anti-Mi-2β, the most common autoantibody seen in dermatomyositis, others, such as anti-SAE, anti-TIF-1γ, and anti-NXP-2, were not tested. The latter two are strongly associated with malignancy [5,6]. The risk of malignancy is increased five to sevenfold in patients with dermatomyositis [7]. Among many malignancies, carcinoma of the gallbladder is a rare cause of dermatomyositis. Histologically, all cases of gallbladder carcinoma associated with dermatomyositis were adenocarcinomas, whereas endocrine paraneoplastic syndromes such as hypercalcemia, hyponatremia, and Cushing’s syndrome are more commonly seen with small-cell gallbladder carcinoma. Myositis autoantigens are markedly expressed in several malignancies known to be associated with dermatomyositis. These cross-react with regenerating muscle fibers, which also express these antigens abundantly, leading to myositis [8]. Risk factors for malignancy in a case of dermatomyositis include older age, presence of dysphagia, cutaneous vasculitis and necrosis, and the presence of capillary damage on muscle biopsy [9]. The first two risk factors were present in our patient. There are no clear guidelines on the extent of screening for malignancy in patients with dermatomyositis. Patients with one or more risk factors should be evaluated with a chest radiograph; stool occult blood; age and sex-appropriate screening tools such as Pap smear, mammography; CT of the abdomen, pelvis, and chest; and colonoscopy, if the above investigations are abnormal. Positron emission tomography, even though very sensitive to detect malignancy, should be reserved for patients deemed to be very high risk, with other investigations being negative [10]. As dermatomyositis can precede malignancy, continued screening is recommended in patients with tumor-specific myositis antibodies or poor response to treatment, at least for one to two years, beyond which the risk of malignancy falls substantially.

## Conclusions

Screening for malignancy should be performed in all cases of dermatomyositis. Older age, presence of dysphagia, and certain myositis-specific antibodies are predictors of malignancy in a case of dermatomyositis. Carcinoma of the gallbladder is a rare cause of dermatomyositis and should be ruled out with appropriate investigations.
